# Smart Capacitive Transducer for High-Frequency Vibration Measurement

**DOI:** 10.3390/s25061639

**Published:** 2025-03-07

**Authors:** Vygantas Augutis, Gintautas Balčiūnas, Pranas Kuzas, Darius Gailius, Edita Raudienė

**Affiliations:** 1 Metrology Institute, Kaunas University of Technology, Studentu Str., 50-454 Kaunas, Lithuania; vygantas.augutis@ktu.lt (V.A.); darius.gailius@ktu.lt (D.G.); edita.raudiene@ktu.lt (E.R.); 2 Department of Electronics Engineering, Kaunas University of Technology, Studentu Str., 50-445 Kaunas, Lithuania; pranas.kuzas@ktu.lt

**Keywords:** auto-calibrating capacitive transducer, capacitive transducer, high-frequency vibration measurement, nanometer displacement measurement

## Abstract

A smart capacitive transducer (SCT) for high-frequency vibration (HFV) measurements was developed, featuring self-calibration for the improvement of measurement accuracy. Measurements using this transducer are performed by positioning it over a thin (10 µm) dielectric layer on a conductive surface. This method was shown to be a non-contact vibration measurement technique for solid surfaces at frequencies over 10 kHz. Auto-calibration is performed every time the SCT is placed on the object being measured. This reduces the influence of positioning and the object’s surface properties on the measurement results. For the transducer’s auto-calibration, a predefined vibration of the measurement electrode is induced. This is achieved using a waveguide excited by a piezo element. The diameter of the developed SCT is 5 mm, with a frequency range of 10 kHz to 1 MHz, an object HFV amplitude measurement resolution of several picometers, and a repeatability error of several percent.

## 1. Introduction

High-frequency vibrations (HFVs) occur in solid bodies when they interact with other solid bodies or with flows. The first case includes all machining processes, such as cutting, grinding, welding, etc. Various friction pairs, ranging from bearings to biological joints, are also included. The second case involves the interaction of solid surfaces with gases, liquids, particles, and electromagnetic fields. In all these scenarios, the frequency of the mechanical vibrations can range from kilohertz to megahertz. Therefore, vibrations with a frequency exceeding 10 kHz are considered HFVs. HFV measurement enables diagnostics and quality control of processes and objects. For example, in the case of acoustic emission, the reliability of an object can be evaluated, and the location of a defect can be determined. Piezoelectric, optical, and capacitive transducers (sensors) are commonly used for HFV measurements [1,2,3,4]. Piezoelectric transducers are characterized by their simplicity, high sensitivity, and ability to operate in complex environments. However, their drawback is the complex evaluation of their frequency characteristics, both during calibration and when measuring the object. This complexity arises from the interaction of the transducer with the calibration stand or the object.

Optical transducers are non-contact; they have no impact on the object’s properties. In terms of HFV measurement, laser interferometers or Doppler vibrometers are the most commonly used optical transducers. They are characterized by the highest precision, broad bandwidth, and maximum dimensional resolution. The main disadvantages are their lower sensitivity and complexity.

Capacitive transducers have intermediate properties. They may be non-contact, sensitive, and accurate, and can also operate in harsh environments [5,6,7]. However, currently available sensors are sensitive to surface irregularities, variations in humidity and temperature, and contamination of interfacial media [8,9]. Additionally, positioning them with respect to an object may be problematic. Furthermore, they are susceptible to electrical interference.

A lot of schematic and design solutions for capacitive transducers are available [10,11,12,13,14,15]. Fixed charge transducers, which are among the best options for HFV measurement, were selected for further investigation. Similarly, to other capacitive transducers, fixed charge transducers face the challenge of precise positioning on the object, which complicates their construction and affects their sensitivity.

The sensitive area of the capacitive transducer is determined by the area of the electrodes and the distance of the separating gap (cavity). In the HFV measurement, the air gap and the electrodes comprise a mechanical resonant system. The theoretical analysis of such a system optimized for pressure measurement was presented and the analytical model was proposed in [16]. The insulator in this work features a cylindrical cavity. The cavity requires precision machining of the insulator and is optimized for pressure measurement in strictly spaced capacitive transducer cases. Such an approach would increase the complexity of the positioning of the transducer in the case of vibration measurement on flat surfaces of various vibrating elements.

The insulator is exposed to wear, dirt, oil, and potential punctures or dents in the bottom part of the cavity membrane. The capacitive transducer positioning problem was addressed in [17]. The mechanical approach of leveling may be applicable in some cases but this method would require additional adjustment of the contact zone, which is impractical in application.

From an application perspective, commercial capacitive HFV transducers feature a flat, sensitive surface [18] with a geometrically formed air gap. The variability of sensitivity due to cavity geometry parameter deflections and deformation during the placement of the capacitive transducer is considered as the main issue. To mitigate the positioning and variability of sensitivity problems, the new approach of forming the sensitive gap was researched by authors of this work. The proposed solution comprises a high-density dielectric layer instead of an air cavity between the object and sensing electrode which, first of all, improves mechanical rigidity and electrical insulation between electrodes. This approach improves the positioning repeatability. The sensitivity variability control upon each placement of the transducer is essential for precise HFV amplitude measurements. For this purpose, the smart principle of auto-calibration is implemented in the proposed transducer using external mechanical impulse source and acoustic waveguide. To the best of the authors’ knowledge, these features have not been previously applied in capacitive HFV transducer implementations. Furthermore, the proposed transducer introduces new functionalities, including the measurement of dielectric material properties. The theoretical principle of operation, influence of the high-density dielectric layer to the proposed transducer frequency response characteristic, noise performance, mechanical measured vibration signal shape comparison with laser interferometer, and repeatability improvement assessment in auto-calibration mode are investigated and presented in this paper.

## 2. Smart Capacitive Transducer Structure and Functioning Theoretical Analysis

**Figure 1:**
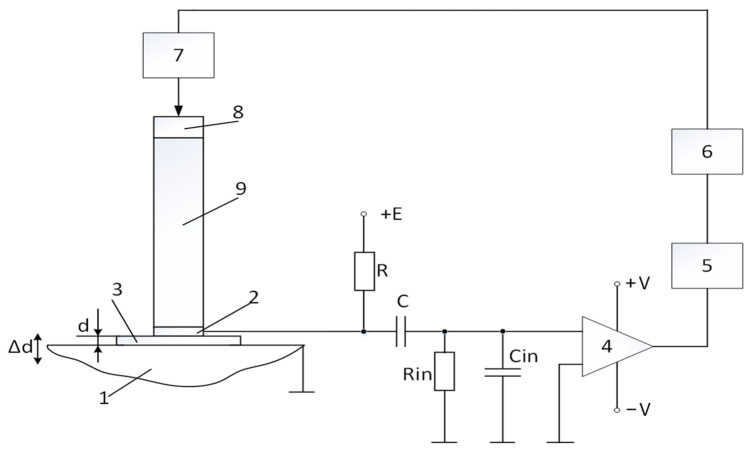
Structural scheme of SCT: 1—measurement object; 2—electrode; 3—dielectric; 4—amplifier; 5—ADC; 6—computer; 7—generator; 8—piezoelectric element; 9—acoustic waveguide.

The structural scheme of the smart capacitive transducer (SCT) is provided in Figure 1.

The application of piezoelectric element 8 and waveguide 9 is one of the main features distinguishing this system from conventional transducers. Additionally, it allows for the measurement of object displacement through dielectric materials (in most measurements, a 10 µm thick dielectric paper was used).

A key aspect of this structure is the distance, *d* (the gap), between the object’s metal surface 1 and electrode 2. Together with dielectric 3, they form an electrical capacitance.

(1) $$C_{x}=\frac{\varepsilon_{r}\varepsilon_{0}A}{d},$$

where *A* is the surface area of the electrode 2; *ε*_0_ ≈ 8.854 × 10^−12^ F/m vacuum dielectric permittivity; *ε_r_* is the dielectric permittivity; and *d* is the distance between object 1 and electrode 2.

Typically, the distance *d* is much smaller than the diameter of electrode 2. In the research conducted on the prototype transducers, the values were *d* ≈ 10^−5^ m; *ε_r_* ≈ 2; *S* ≈ 2.5 × 10^−5^ m^2^. In this particular case, the capacitance *C_x_* ≈ 44 pF. The transducer’s primary signal caused either by vibrations of the measurement object or by an auto-calibration impulse is generated in its active zone. Further, theoretical analysis of the transducer active zone is presented. The capacitance *C_x_* of the transducer is charged according to the following equation:

(2) $$Q=EC_{x}.$$

The charge in the capacitance *C_x_* remains unchanged if the surface of the object vibrates with an amplitude ∆*d* << *d*, the capacitance *C* >> *C_x_*, and the frequency of the object’s surface vibration *f* satisfies the conditions outlined in Equation (3).

(3) $$f>\frac{1}{2\pi \;(C_{x}+C_{in})(R||R_{in})}\;,$$

In this case, only the voltage changes (where *C_in_* and *R_in_* are the input capacitance and the resistance of the amplifier, and *R||R_in_* represents the parallel combination of *R_in_* and *R* resistance). From Equation (2), after logarithmic transformation and differentiation, the voltage variation corresponds to the variation ∆*C_x_* and is expressed by Equation (4).

(4) $$\Delta U=-E\frac{\Delta C_{x}}{C_{x}}$$

From Equation (1), it follows that, in the case of constant *ε_r_*, *ε_0_*, and *S*, the following occurs:

(5) $$\frac{\Delta C_{x}}{C_{x}}=-\frac{\Delta d}{d}$$

Then, considering that *C >> C_x_* and *C_in_* can be considered parallel to *C_x_*, from Equations (4) and (5), we obtain the following:

(6) $$\Delta U=E\frac{\Delta d}{d}\frac{C_{x}}{C_{x}+C_{in}}.$$

The computer registers the digitized voltage.

(7) $$U=\Delta UK_{A}K_{D},$$

where *K_A_* is the amplifier gain, and *K_D_* is the ADC (analog-to-digital converter) transfer coefficient.

From Equations (5)–(7), we obtain that the measured value equals the following:

(8) $$\Delta d=\frac{d}{EK_{A}K_{D}}\left(1+\frac{C_{in}}{C_{x}}\right)U=KU,$$

where *U* is the computer-recorded voltage and *K* is a constant coefficient.

Expression (8) shows that the measurement result depends on *d* and *C_x_*, which may vary each time when the capacitive transducer is placed on the object. This issue can be addressed by calibrating the transducer after each placement. Calibration is performed by applying a known displacement ∆*d*_0_ to the measurement electrode, which is considered equivalent to the surface displacement of object 1.

Displacement is generated according to the block diagram in Figure 1: computer 6 sends a control pulse to generator 7, which excites piezoelectric element 8. This generates waves that propagate along waveguide 9, causing displacement ∆*d*_0_ of electrode 2. The waveguide is necessary to separate the generator’s electric pulse in time from the vibration of the electrode, preventing interference during the measurement of the reference displacement ∆*d*_0_ in the calibration of the transducer.

The calibration procedure involves evaluating the displacement ∆*d*_0_ using a laser interferometer. Once ∆*d*_0_ is determined, it can serve as a reference for subsequent measurements, provided the excitation conditions remain constant.

When the transducer is placed over a dielectric material (dielectric paper in this case), the transmission coefficient is calculated using Equation (9).

(9) $$K=\frac{{\Delta d}_{0}}{U_{0}},$$

where, *U*_0_ is the input signal recorded by computer 6, corresponding to the reference displacement ∆*d*_0_.

If the object’s surface is free of additional vibrations, a single measurement of ∆*d*_0_ is sufficient to determine the transmission coefficient *K*. However, if the surface is vibrating and these vibrations do not correlate with ∆*d*_0_, averaging several measurements can help mitigate the influence of unrelated vibrations.

Once *K* is determined, the displacement of the object ∆*d_x_* is evaluated using Equation (10).

(10) $$\Delta d_{x}=KU_{x}.$$

where *U_x_* is the recorded signal waveform proportional to the object’s surface displacement, and ∆*d_x_* is the measured object surface displacement obtained with the capacitive transducer.

## 3. Evaluation of Acoustic Contact Influence on Measurement Result

**Figure 2:**
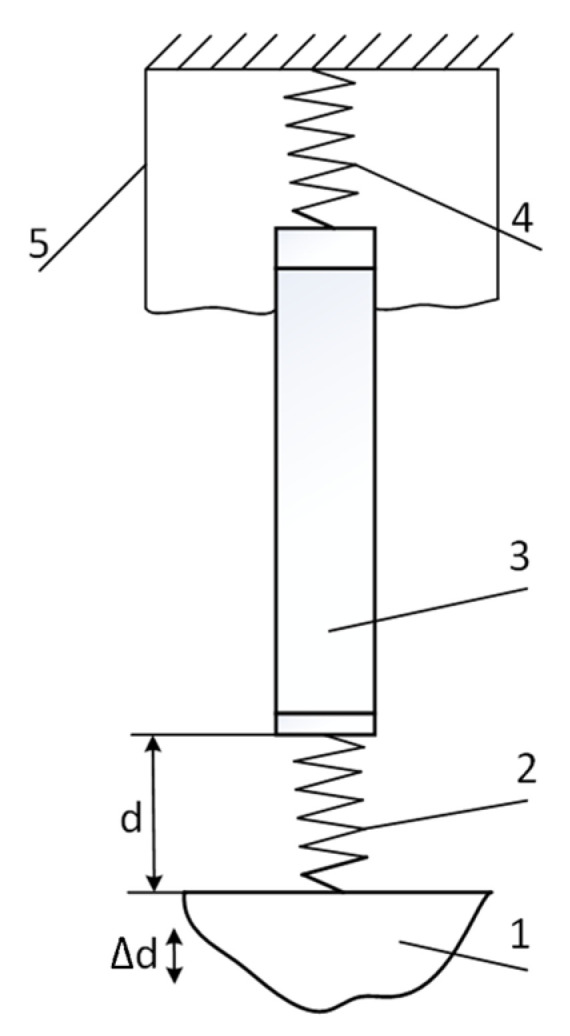
SCT mechanical model: 1—vibrating object; 2—dielectric (equivalent to spring of stiffness *S_1_*); 3—inertial mass *m* (including electrode, waveguide, and piezoelectric element); 4—clamping spring with stiffness *S_2_*; 5—housing.

If the condition in Equation (3) is not met, the dynamic properties of the measurement system may affect the measurement results. At certain frequencies, a resonance related to the mechanical properties of the measurement system may be observed. This resonance can be analyzed by examining the mechanical model of the transducer presented in Figure 2.

We have a classic model system described by a second-order differential equation. The resonant frequency of such a system is given by the following:

(11) $$f_{rez}=\frac{1}{2\pi }\sqrt{\frac{S}{m}},$$

where *S* is the stiffness and *m* is the moving mass.

**Figure 3:**
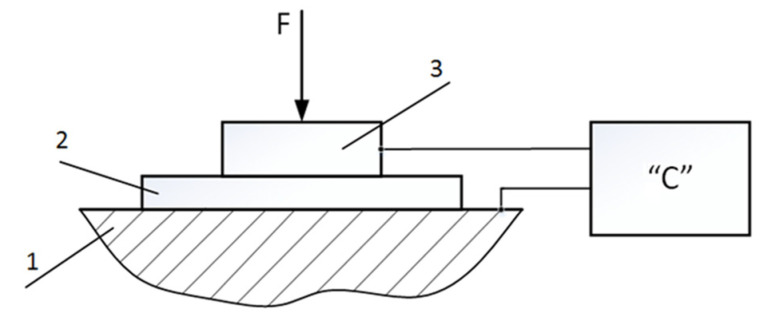
Stiffness measurement scheme: 1—base; 2—dielectric (paper); 3—electrode; “C”—electrical capacitance meter.

Since the stiffness of the dielectric material is substantially higher than that of the clamping string, we approximate *S ≈ S*_1_. This stiffness and mechanical quality can be determined experimentally using the structural scheme shown in Figure 3.

**Figure 4:**
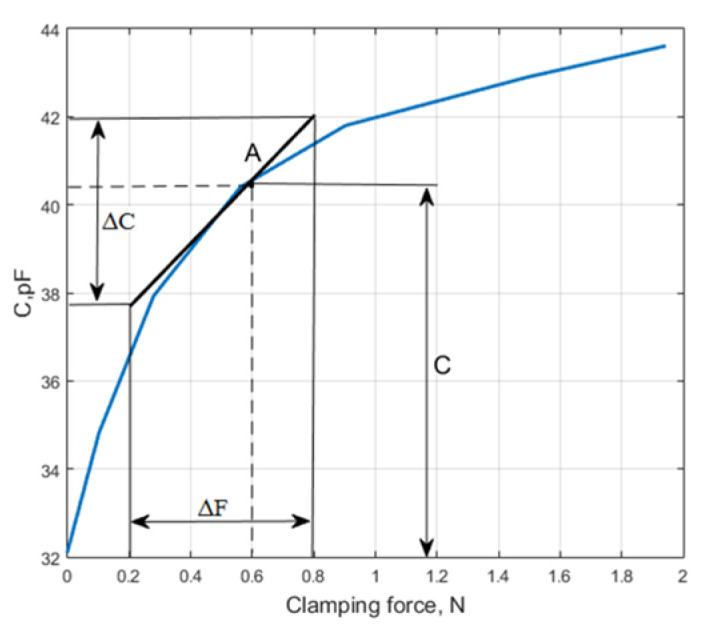
Dielectric material stiffness measurement graph.

Measurement results, obtained using the prototype capacitive transducer, are presented in Figure 4.

From Equation (5), we have the following:

(12) $$\Delta d=d\frac{-\Delta C}{C}.$$

In this particular case, the initial thickness of the dielectric material is *d* ≈ 10 µm, and the vibrating mass is *m* = 0.0025 kg. The ratio ∆*C/C* and the change in force under a selected clamping force at point A are obtained from the graph in Figure 4: *C* = 40.3 pF, ∆*F* = 0.6 N, ∆*C* = 4.2 pF.

Using these data, the stiffness *S*_1_ is calculated as follows:

(13) $$S_{1}=\frac{\Delta F}{\Delta d}=\frac{\Delta F}{d}\frac{C}{\Delta C}=5.76\times 10^{5}\frac{N}{m}.$$

The resonant frequency is determined using a 2.5 g inertial mass.

(14) $$f_{rez}=\frac{1}{2\pi }\sqrt{\frac{S_{1}}{m}}=2.42\times 10^{3}\;Hz.$$

**Figure 5:**
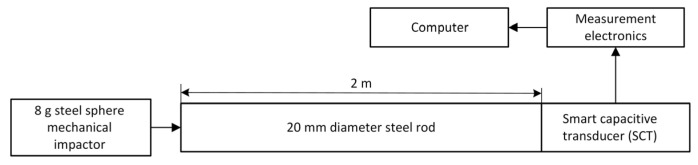
SCT free mechanical vibration measurement block diagram.

An experimental study of the interaction between the SCT and the object was conducted according to the diagram shown in Figure 5.

For this purpose, pulse displacement was applied to the surface of the object. The pulse duration was approximately 50 µs. The pulse was generated using a 2 m long, 20 mm diameter steel rod.

**Figure 6:**
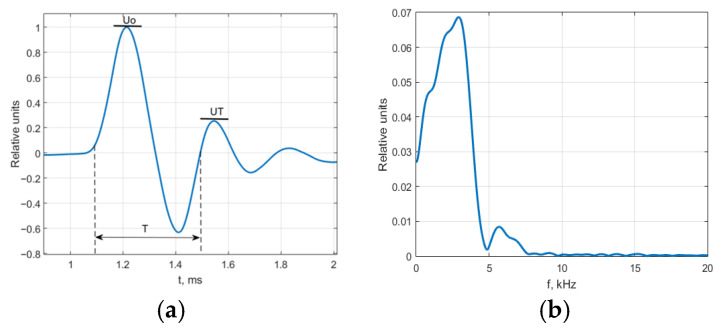
Free mechanical vibrations of capacitive transducer measurement electrode: (**a**) measured signal; (**b**) calculated frequency response.

The SCT was positioned at one end of the rod, while the other end was struck with an 8 g steel sphere. The resulting SCT pulse response is shown in Figure 6a, and its frequency response is provided in Figure 6b.

According to Figure 6, it can be observed that at frequencies above 5 kHz, there is no significant interaction between the object and the SCT. Thus, at high frequencies, the SCT can be considered contactless, benefiting from its positioning through the dielectric material.

## 4. Evaluation of Metrological Characteristics

A Polytec laser interferometer OFV-512 was used for the calibration of the SCT. The main characteristics of the interferometer are as follows:

- Frequency range: 5 kHz–1 MHz.
- Sensitivity: 2 µm/V.
- Accuracy: 2%.

**Figure 7:**
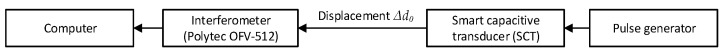
SCT reference displacement measurement with interferometer.

**Figure 8:**
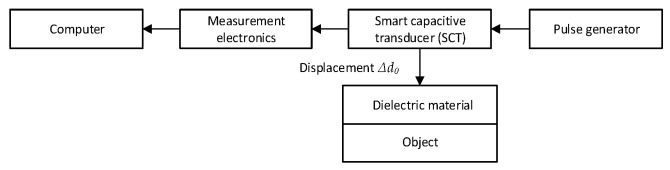
SCT reference displacement measurement using SCT schematic block diagram.

The measurement task was to evaluate the displacement ∆*d*_0_. Reference displacement measurements were conducted according to the setup shown in Figure 7. Auto-calibration measurement setup for the proposed SCT, is shown in Figure 8.

**Figure 9:**
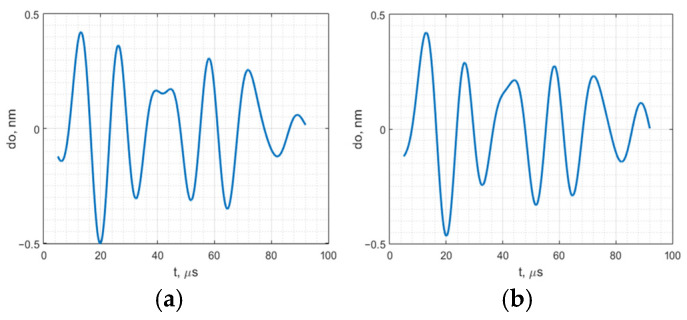
Measured SCT reference displacement waveform: (**a**) measured with interferometer; (**b**) measured with SCT internal schematics.

The same vibration was measured using the capacitive transducer placed on a stationary object over a 10 µm thick dielectric material. The reference displacement waveform measured using interferometer is presented in Figure 9a. The resulting signal measured by the proposed SCT is shown in Figure 9b. 

Figure 9 demonstrates that in both cases, the measured displacement waveforms remained almost unchanged, with a correlation coefficient *ρ* = 0.975. This indicates that, within the relevant frequency range, the interaction between the SCT and the object is negligible.

The main metrological characteristics of the SCT include the measurement vibration frequency range, resolution, and error.

The lower limit of the measurement frequency is determined by the mass of the moving part of the SCT and the stiffness of the clamped dielectric material. The minimum measurable frequency can be approximately calculated using Equation (15).

(15) $$f_{min}\approx \left(3\;to\;5\right)\times f_{rez},$$

where *f_rez_* is calculated from Equation (11).

The measurement bandwidth is limited by the electronic components of the measurement system and can exceed 1 MHz.

The resolution is influenced by electrical noise and depends on factors such as the amplifier components, the sizes of the capacitances and resistors, and the frequency range.

**Figure 10:**
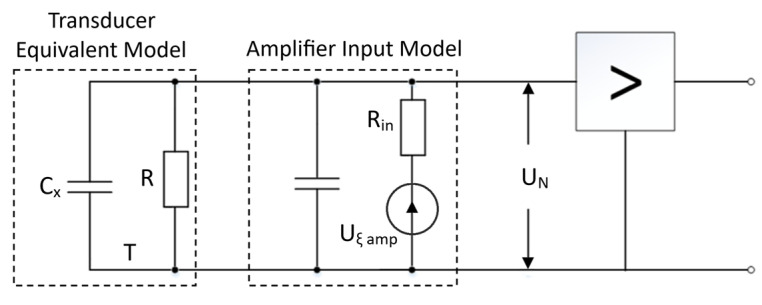
Noise model.

To evaluate the amplifier noise, an equivalent circuit model was created based on the Figure 1 schematic and is shown in Figure 10.

The thermal noise of an RC circuit is as follows:

(16) $$U_{CN}=\sqrt{\frac{kT}{C},}$$

where *k* is the Boltzmann constant (1.38 × 10^−23^ J/K); and *T* is the absolute temperature (*T* = 300 K).

*C* = *C_x_* + *C_in_*, *C* = 40 pF.

The noise of the low-noise amplifier is characterized by the input FET noise *U_N_*, which is approximately *U_ξ_* = 0.7 nV/$\sqrt{Hz}$. The equivalent noise bandwidth for the amplifier is given by $∆F_{e}=\frac{\pi }{2}f_{max}$. Therefore, the RMS noise at the amplifier’s input can be calculated as follows:

(17) $$U_{N}=\sqrt{\frac{kT}{C}+{U_{\xi }}^{2}\left(\frac{\pi }{2}f_{max}\right).}$$

At the above values and *f_max_* = 1.6 MHz, we have *U_N_* ≈ 10^−5^ V.

We assume that the noise distribution is Gaussian. With a probability of 0.95, the noise falls within the range of 4·*U_N_*. From Equation (6), we have the following:

(18) $$\Delta d=\frac{\Delta Ud\left(C_{X}+C_{in}\right)}{{E\;C}_{X}}\;,$$

Equating ∆*U* = 4*U_N_* = 4 × 10^−5^ V, with *E* = 200 V, *d* = 10^−5^ m, and *C_in_ << C_x_*, Equation (18) yields a discrimination threshold of ∆*d* ≈ 2 pm.

It is noteworthy that some significant factors were not evaluated here. Firstly, if the frequency of the variable component of the voltage *E* falls within the frequency band of the measured signals, it should be added to the noise voltage *U_N_*. Additionally, since we are dealing with high-impedance circuits, alternating current and voltage interferences are possible.

On the other hand, resolution can be improved through averaging if the measured vibrations are consistent and repeatable.

**Figure 11:**
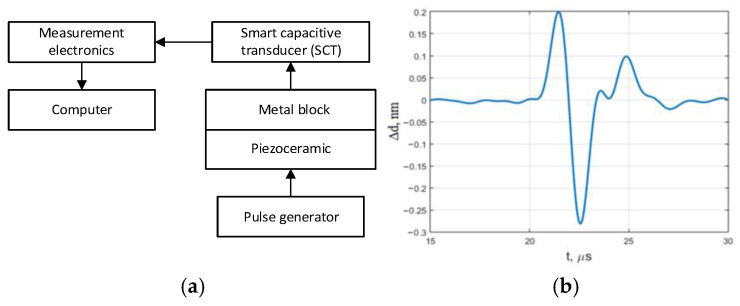
Surface vibration of block measurement: (**a**) measurement block diagram; (**b**) measured waveform.

To evaluate the efficiency and measurement uncertainty of auto-calibration, repeated measurements were performed. A steel block with a thickness of 140 mm was used for this purpose. Vibration was excited on one side of the block using a piezoelectric transducer, and measurements were conducted on the other side using the SCT, as shown in Figure 11a. The measured vibration waveform is presented in Figure 11b.

The following three types of measurements were performed:

- Calibration of the SCT was conducted before each measurement.
- Calibration was performed once using a 20 µm thick dielectric layer between the capacitive transducer measurement electrode and the block surface.
- The dielectric material thickness was changed to 10 µm while maintaining the previously obtained transmission coefficient with the 20 µm layer.

All types of measurements were repeated 20 times, each time removing and repositioning the SCT on the metal block. The measured parameter was the double amplitude of the pulse ∆*d_p-p_*. The results, i.e., the measured peak-to-peak amplitudes, are shown in Table 1.

As expected, when calibration is performed with a 20 µm dielectric material layer and measurements are carried out with a 10 µm dielectric layer, the measured displacement amplitude changes. This demonstrates that the proposed measurement method, which uses a dielectric material between the measurement electrode and the object, requires auto-calibration. It is also evident that repeated calibration after each reapplication of the SCT reduces the standard deviation (*σ*) and mitigates the impact of the dielectric layer thickness.

## 5. Conclusions

The proposed SCT for HFV measurement possesses the distinct features: a high-density dielectric layer of *d* ≈ 10 µm thickness and implemented auto-calibration with a piezoelectric element separated by an acoustic waveguide. The proposed structure enables improved repeatability and smart auto-calibration after positioning the transducer on the object. The theoretical principle of operation was presented and the main influencing factors were mathematically assessed.

The acoustic contact of the sensitive zone of the SCT was investigated in the 0–20 kHz frequency range. It was determined by the free mechanical vibration measurement method that the capacitive transduced measurement electrode possesses no significant interaction between the object and the SCT at frequencies above 5 kHz. This determines the lowest frequency limit of HFV measurable by the proposed SCT with increased error. The interaction is negligible at HFV frequencies above 10 kHz.

The reference displacement waveform was measured with a Polytec laser interferometer OFV-512 and SCT. A good coherence between the HFV waveforms was achieved with a correlation coefficient ρ = 0.975.

The SCT repeatability performance was assessed. The variability of the measurement result was registered using a reference HFV mechanical signal of 0.480 nm peak-to-peak-amplitude in three modes of measurement: by implementing auto-calibration before each of 20 measurements, by implementing auto-calibration once before series of 20 measurements, and by swapping the dielectric material (from *d* = 20 µm to *d* = 10 µm), keeping the same transmission coefficient established at *d* = 20 µm. It was experimentally determined that at *d* = 20 µm, the measured impulse amplitude was 0.496 nm using the same transmission coefficient for the series of measurements and 0.478 nm, accordingly with auto-calibration before each measurement. At *d* = 10 µm, the measured average amplitude increased 1.58 times using the same transmission coefficient for the series of measurements. It remained close to the reference value with auto-calibration before each measurement. The standard deviation also reduced significantly using auto-calibration. Calibration after each application of the SCT to the measurement object reduces the effects of dielectric material and electrode geometry.

The noise influence was assessed. The estimated thermal noise contribution to the measurement error discrimination threshold is ∆*d* ≈ 2 pm. Thus, using the SCT with a 5 mm diameter electrode, resolution of several picometers may be reached in the frequency range from 10 kHz to 1 MHz. The proposed SCT in auto-calibration mode is also suitable for determining the properties of high-density dielectric materials including density, dielectric constant, and thickness.

## Figures and Tables

**Table 1:** Experimental assessment of SCT repeatability in different smart auto-calibration modes.

	Calibration Before First Measurement with 20 µm Thickness Dielectric Material		Calibration Before Each Measurement	
Thickness of dielectric d, µm	20	10	20	10
*S_A_*, nm	0.496	0.787	0.478	0.481
*σ*, nm	0.03	0.054	0.016	0.009

*S_A_*—average; *σ*—standard deviation.

## Data Availability

The data supporting the findings of this study are not available.
